# Supplementary material for: Comparative metabolomics with Metaboseek reveals functions of a conserved fat metabolism pathway in C. elegans
Source: Nat Commun. 2022 Feb 10;13:782. doi: 10.1038/s41467-022-28391-9 (PMC8831614; doi:10.1038/s41467-022-28391-9)
Supplement: Supplementary file 1 — Supplementary Information [file 41467_2022_28391_MOESM1_ESM.pdf]

Comparative metabolomics with Metaboseek reveals functions of a conserved fat metabolism pathway in *C. elegans*

Supplementary Information

Maximilian J. Helf[1], Bennett W. Fox[1], Alexander B. Artyukhin[2], Ying K. Zhang[1], Frank C. Schroeder[1,*]

[1]Boyce Thompson Institute and Department of Chemistry and Chemical Biology, Cornell University, Ithaca, New York 14853, United States, [2]Chemistry Department, College of Environmental Science and Forestry, State University of New York, Syracuse, New York 13210, United States

These authors contributed equally: Maximilian J. Helf, Bennett W. Fox

*Correspondence to fs31@cornell.edu

Table of Contents

This file includes Supplementary Figures 1-17, Supplementary Methods, and Supplementary Tables 1-4.

Supplementary Figures 3
Supplementary Methods 21
Supplementary Tables... 23

Supplementary Figures

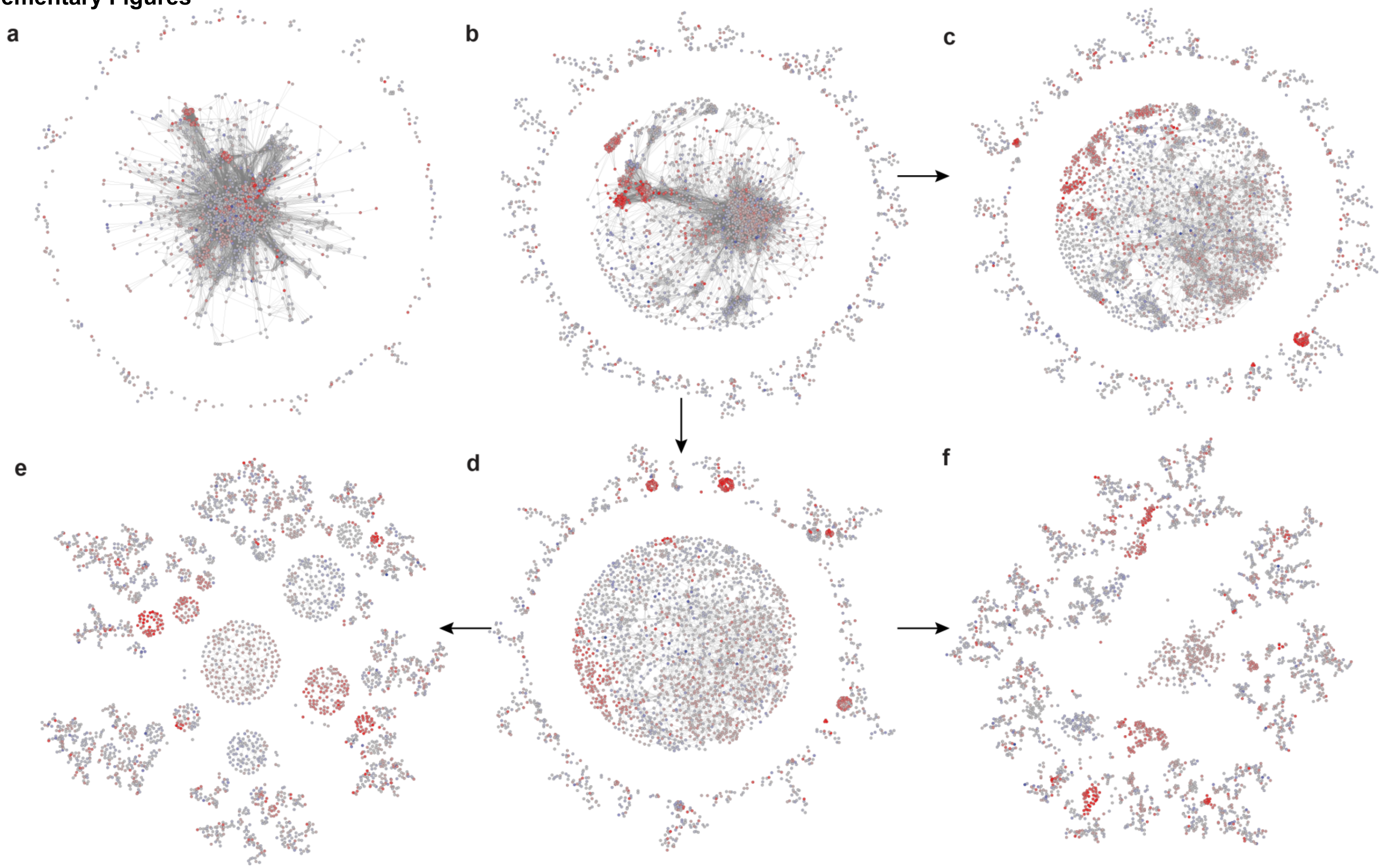

Supplementary Figure 1. Modifying MS/MS networks with the *Simplify Network* function. **a,** Negative ion MS/MS network constructed using a similarity score threshold of 0.6 and requiring a minimum of two matching fragments. **b,** Negative ion MS/MS network constructed using a similarity score threshold of 0.6 and requiring a minimum of four matching fragments. **c,** Simplification of the network in (**b**) by restricting the number of edges per node to the top 20, ranked by similarity score. **d,** Simplification of the network in (**b**) by restricting the number of edges per node to the top 10, ranked by similarity score. **e,** Further simplification of the network in (**d**) by restricting the maximum cluster size to 200 nodes, using the Metaboseek default display setting. This MS/MS network was rearranged slightly to save space and is featured in the main text (see **Figure 4**). **f,** Simplification of the network as in (**e**), but using the Metaboseek *KK* display setting.

a

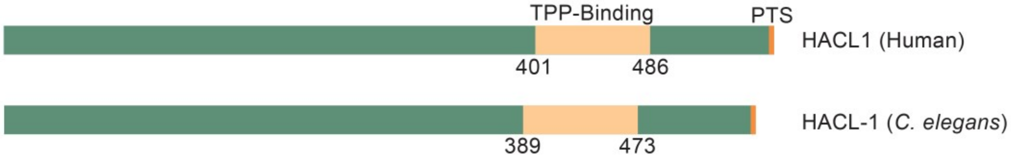

b

```
Human      MPDSNFAERSEEQVSGAKVIAQALKTQDVEYIFGIVGIPVTEIAIAAQQLGIKYIGMRNE
C. elegans -------------MDGASITAAALKSQGVEYMFGVVGFPVIEVGMAAQAHGIKYIGCRNE
                        :.**.: * ***:*.***:**:**:** *:.:***  ****** ***

Human      QAACYAASAIGYLTSRPGVCLVVSGPGLIHALGGMANANMNCWPLLVIGGSSERNQETMG
C. elegans QAAAYAAQAMGYLTGKPVALLVVSGPGILHAIGGLANATVNCWPVVCIGGTADVDLENRG
           ***.***.*:****.:*  . *******::**:**:***.:****:: ***::: : *. *

Human      AFQEFPQVEACRLYTKFSARPSSIEAIPFVIEKAVRSSIYGRPGACYVDIPADFVNLQVN
C. elegans AFQEWSQQESVRNSCKHVSRPTSLHTIPAHIEKAVRCAMYGRPGAVYVDLPGNLVLTSTE
           ****: * *: *    *. :**:*:.:**  ******.::****** ***:*.::*  ..:

Human      VNSIKYMERCMSPPISMAETSAVCTAASVIRNAKQPLLIIGKGAAYAHAE-ESIKKLVEQ
C. elegans EEISFPPQVPLPAPVSIPPIAEIEKAIDTLKSAKKPLVIVGKGAAWSERGATQVQQFLTK
            :     :  :   *:*:   : : .* ..::.**:**:*:*****::.    .::::: :

Human      YKLPFLPTPMGKGVVPDNHPYCVGAARSRALQFADVIVLFGARLNWILHFGLPPRYQPDV
C. elegans SKLPWLATPGGKGVASDLHPRFIGQARSLALREADTVFLIGARFNWILHFGLPPRFQKDV
            ***:* ** ****. * **  :* *** **: **.:.*:***:***********:* **

Human      KFIQVDICAEELGNNVKPAVTLLGNIHAVTKQLLEELDKTPWQYPPESKWWKTLREKMKS
C. elegans KVVQIDICPEEFHQNVKTEVPLLGDIGETLAELTPRL--GDWTYDESTEWFKKLRENAEK
           *.:*:*** **: :***  * ***:*  .  :*  .*    * *  .::*:*.***: :.

Human      NEAASKELASKKSLPMNYYTVFYHVQEQLP-RDCFVVSEGANTMDIGRTVLQNYLPRHRL
C. elegans NRAAVEKFVDDHSTPLNYYAAYQPIREFLANNDVIVINEGANTMDIGRTMMPSRLPKRRL
           *.** :::...:* *:***:.:  ::* *  .* :*:.***********:: . **::**

Human      DAGTFGTMGVGLGFAIAAAVVAKDRSPGQWIICVEGDSAFGFSGMEVETICRYNLPIILL
C. elegans DAGTFGTMGVGHGFSLAAALWARDHSPKTKVLVVQGDSAFGFSAMELETIARYNLPVVTV
           *********** **::***: *:*:**   :: *:********.**:***.*****:: :

Human      VVNNNGIYQGFDTDTWKEMLKFQDATAVVPPMCLLPNSHYEQVMTAFGGKGYFVQTPEEL
C. elegans IINNSGIYRGLLPEDDKAI--EGDRTLALPVLSLTAECRYEEMCKAFGGAGTVVRTVPEI
           ::**.***:*:  :  * :    * * .:* :.*  :.:**:: .**** * .*:*  *:

Human      QKSLRQSLADTTKPSLINIMIEPQATRKAQDFHWLTRSNM 578
C. elegans KAALEKAFQKTDGPTVINALISTDSERKPQADHWLTRSKM 563
           : :*.::: .*  *::** :*. :: ** *  ******:*
```

Supplementary Figure 2. *C. elegans hacl-1 (*formerly *B0334.3*) encodes a protein similar to human HACL1. **a,** Protein structure schematic of human HACL1 and *C. elegans* HACL-1. Both proteins contain C-terminal TPP-binding domains and a peroxisomal targeting signal. **b,** Basic local alignment search tool (BLAST) comparison of human HACL1 and *C. elegans* HACL-1. Proteins are 49% identical overall, 64% identical in the annotated TPP-binding domain (highlighted) and share a type 1 peroxisomal targeting signal (boxed). Asterisks (*) indicate identical, two dots (:) indicates very similar, and one dot (.) indicates somewhat similar amino acid composition.

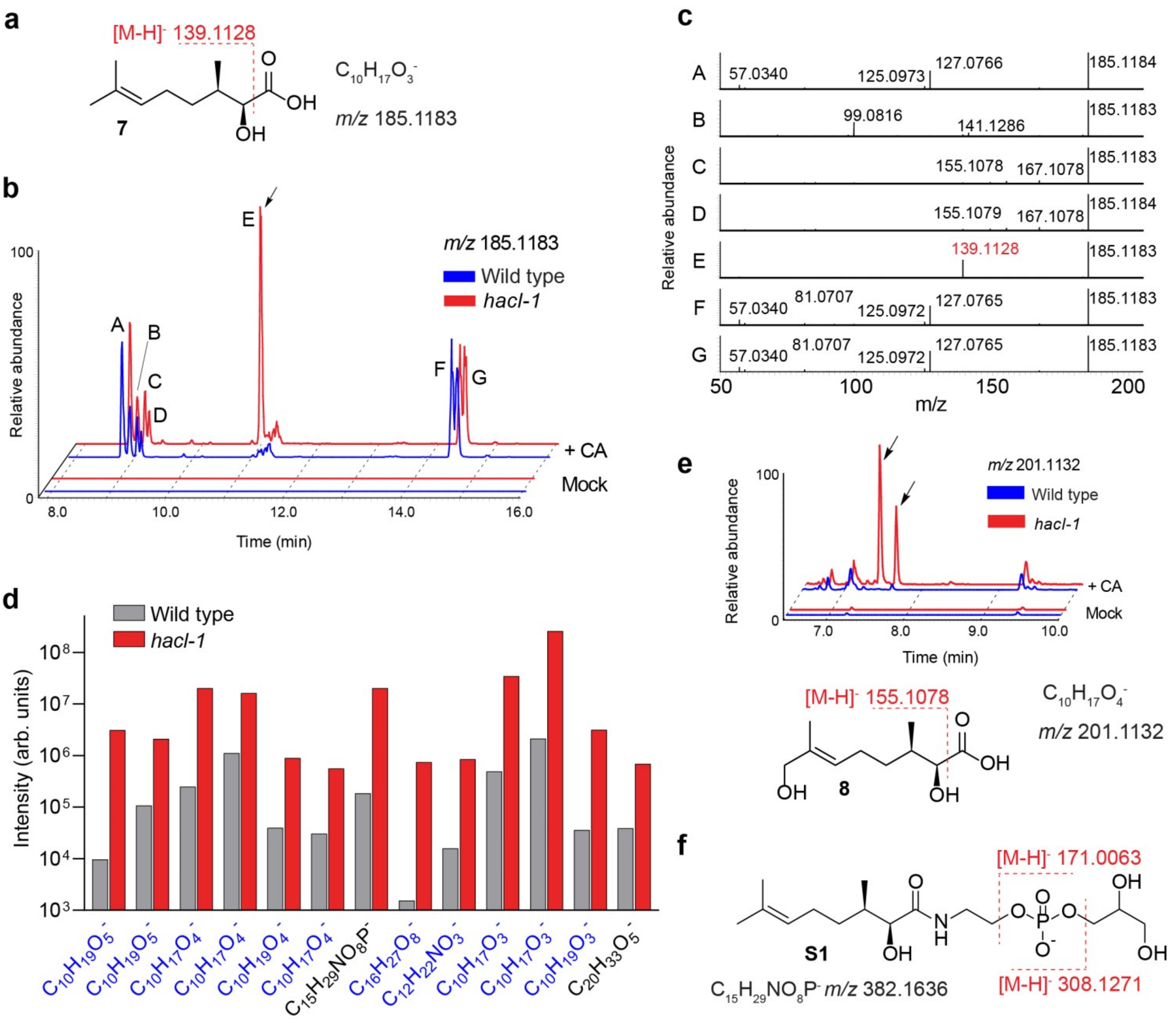

Supplementary Figure 3. Accumulation of α-hydroxy citronellic acid derivatives in *hacl-1*. **a**, Proposed structure of shunt metabolite **7** predicted to accumulate in *hacl-1* following CA supplement. **b**, EIC for *m/z* 185.1183 ($C_{10}H_{17}O_3^-$) reveals seven distinct CA-dependent metabolites (labeled A-G), but only feature E is enriched in *hacl-1* as compared to WT. **c**, MS/MS spectra for metabolites A-G. MS/MS fragmentation of E in negative ion mode produces a strong product ion with *m/z* 139.1128, corresponding to neutral loss of formic acid. **d**, Quantification of CA-derived metabolites in *hacl-1* that are at least $5x10^5$ intensity and 10-fold enriched in *hacl-1* as compared to wildtype animals, organized by increasing RT. Data represent one experiment. Metabolites in blue exhibit fragmentation between the carbonyl- and α-carbon in MS/MS. **e**, EIC for *m/z* 201.113 ($C_{10}H_{17}O_3^-$) reveals several distinct CA-dependent metabolites, two of which are enriched in *hacl-1* (marked with arrows). Both of these metabolites lose formic acid by MS/MS, proposed representative structure **8** below. **f**, Proposed structure and major MS/MS fragmentation reactions for CA-derived *N*-acyl glycerophosphoethanolamide (**S1**) accumulating in *hacl-1* larvae. Source data are provided as a Source Data file.

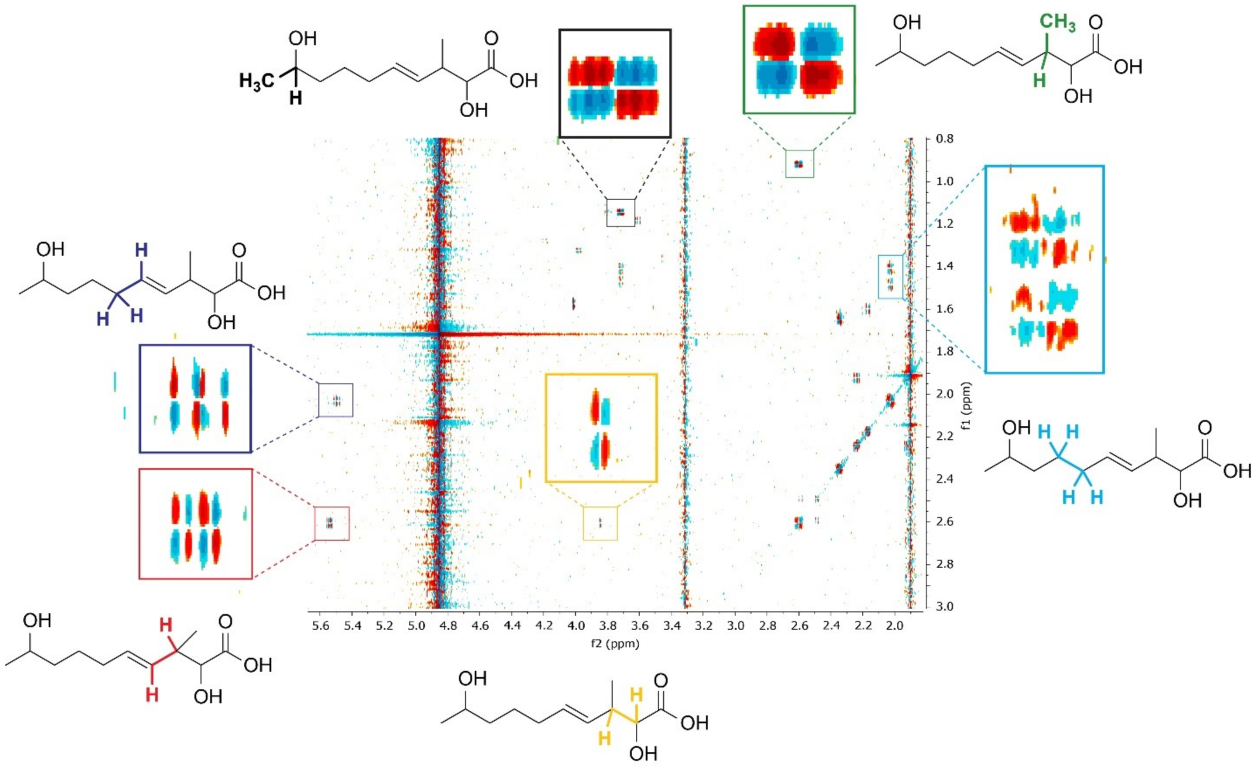

Supplementary Figure 4. Structure elucidation of bemeth#3.1. 2D NMR spectroscopic characterization of an isolated sample of compound **12** (bemeth#3.1, $C_{11}H_{20}O_4$) via dqfCOSY. Shown are relevant dqfCOSY cross peaks and the corresponding structural features.

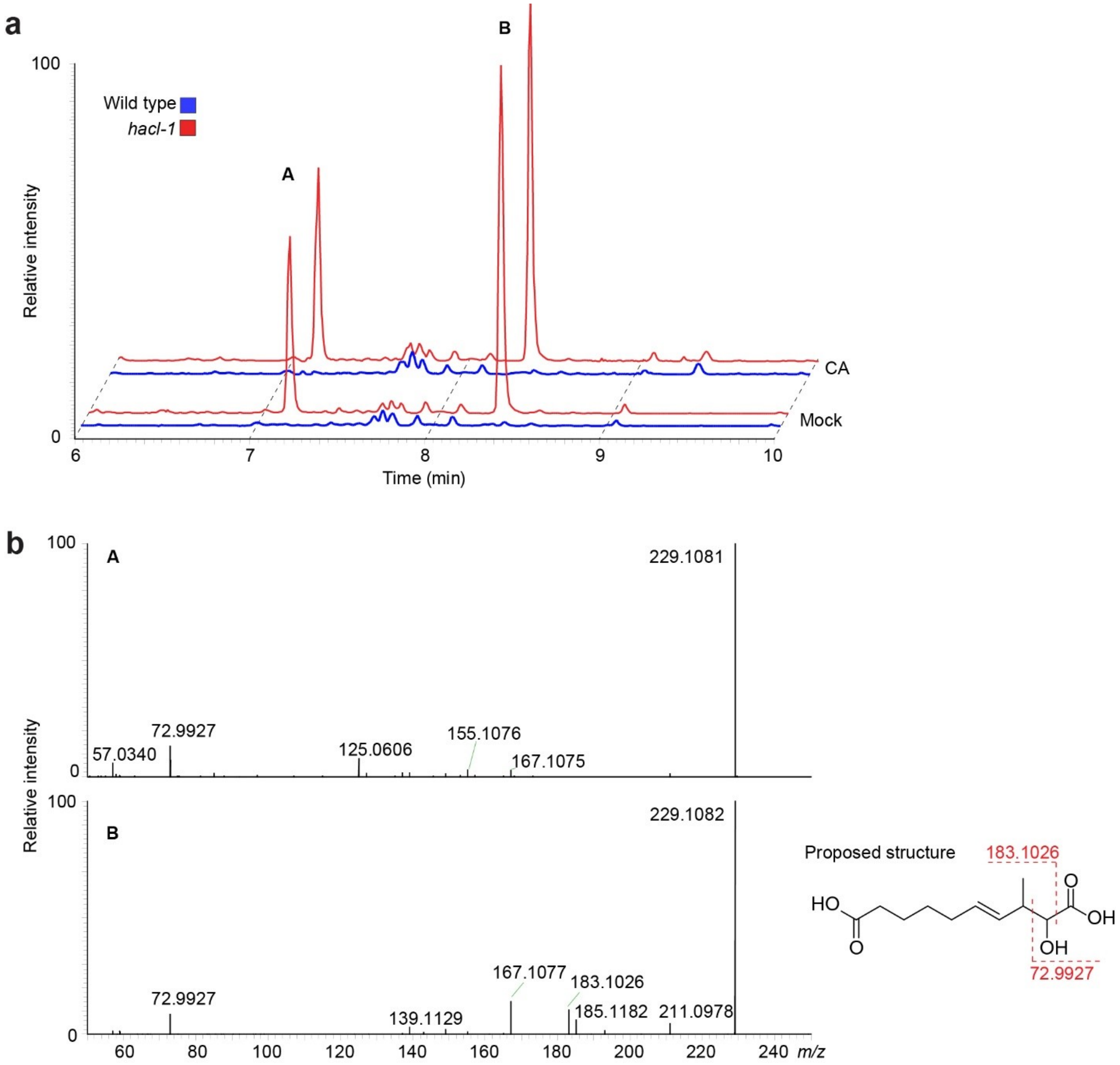

Supplementary Figure 5. Oxidized C_{11} derivatives accumulate in *hacl-1*. **a**, Representative HPLC-MS (negative ion) EIC for *m/z* 229.1082, corresponding to $C_{11}H_{17}O_5^-$, from WT and *hacl-1* animals supplemented with CA or vehicle only, as indicated. Two major isomers are enriched in *hacl-1* irrespective of CA supplement, labeled A and B. **b**, MS/MS spectra for isomers of $C_{11}H_{17}O_5^-$, as indicated in panel (a). Features A and B both exhibit the diagnostic glyoxylate product ion (*m/z* 72.993), but only feature B also exhibits neutral loss of formic acid (*m/z* 183.1026). Proposed structure for B shown.

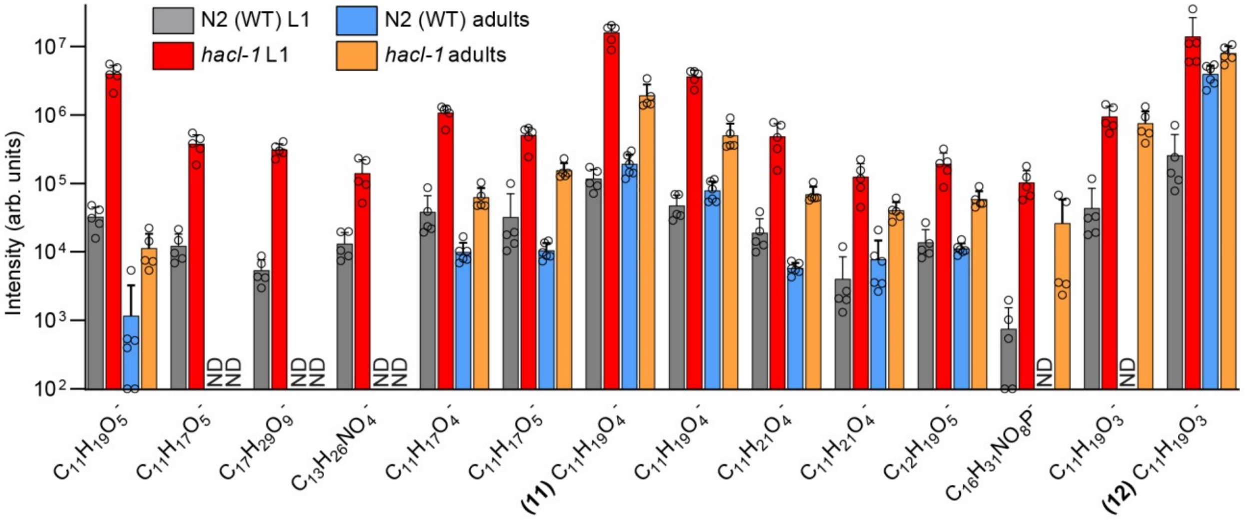

Supplementary Figure 6. Analysis of C_{11} fatty acids in *C. elegans* larvae (L1) and adults. Quantification of metabolites in *exo*-metabolome extracts of synchronized N2 (WT) and *hacl-1* animals from the indicated stage. Quantified metabolites were originally identified as enriched in *hacl-1* L1 larvae *exo*-metabolome extracts (see **Figure 3**). Data for larvae represent five independent experiments and for adults represent six (N2) or five (*hacl-1*) samples from three biologically independent experiments and bars means ± standard deviation. Source data are provided as a Source Data file.

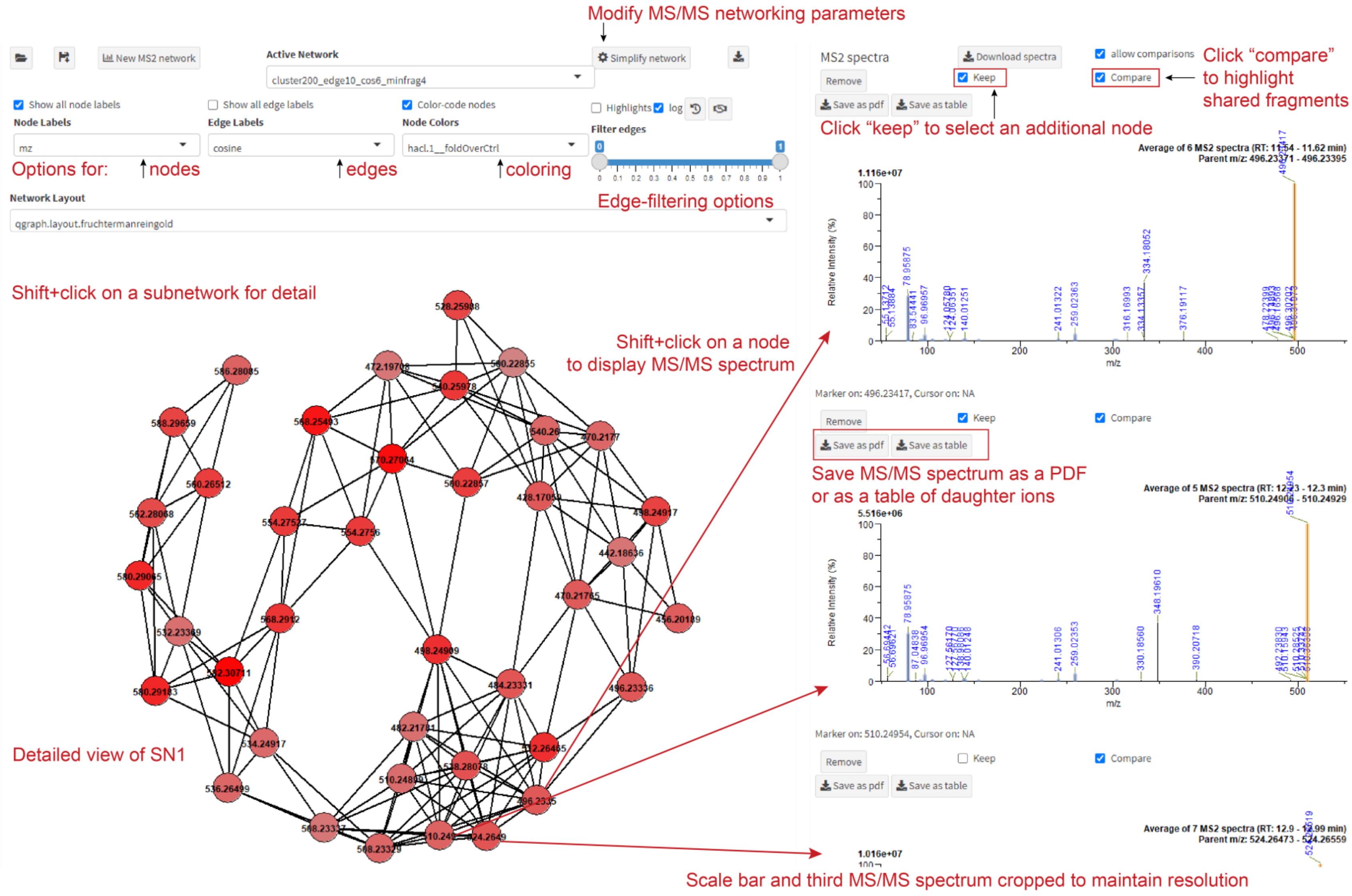

Supplementary Figure 7. Interact with MS/MS data using the *Keep and Compare* function. A screenshot of the *Compare MS2* module within the *Data Viewer*. Shift+click a subnetwork of interest for interactive capability, SN1 shown. MS/MS spectra displayed for *m/z* 496.2341, 510.2495, and 524.2652, corresponding to *N*-acyl GPE-13:1, -14:1, and -15:1, respectively. Toggling the *Keep* button allows users to display multiple MS/MS spectra by clicking additional nodes. Toggling the *Compare* button highlights the parental *m/z* in yellow and any shared fragments are highlighted in blue. MS/MS spectra are interactive: shift+click an *m/z* of interest to display all other *m/z* relative to selection. Neutral loss of a hexose moiety (-162.053) yields the major product ion in each spectrum. MS/MS spectra can be downloaded using the *Save As PDF* function; product ion tables can be downloaded in text format using the *Save As Table* function.

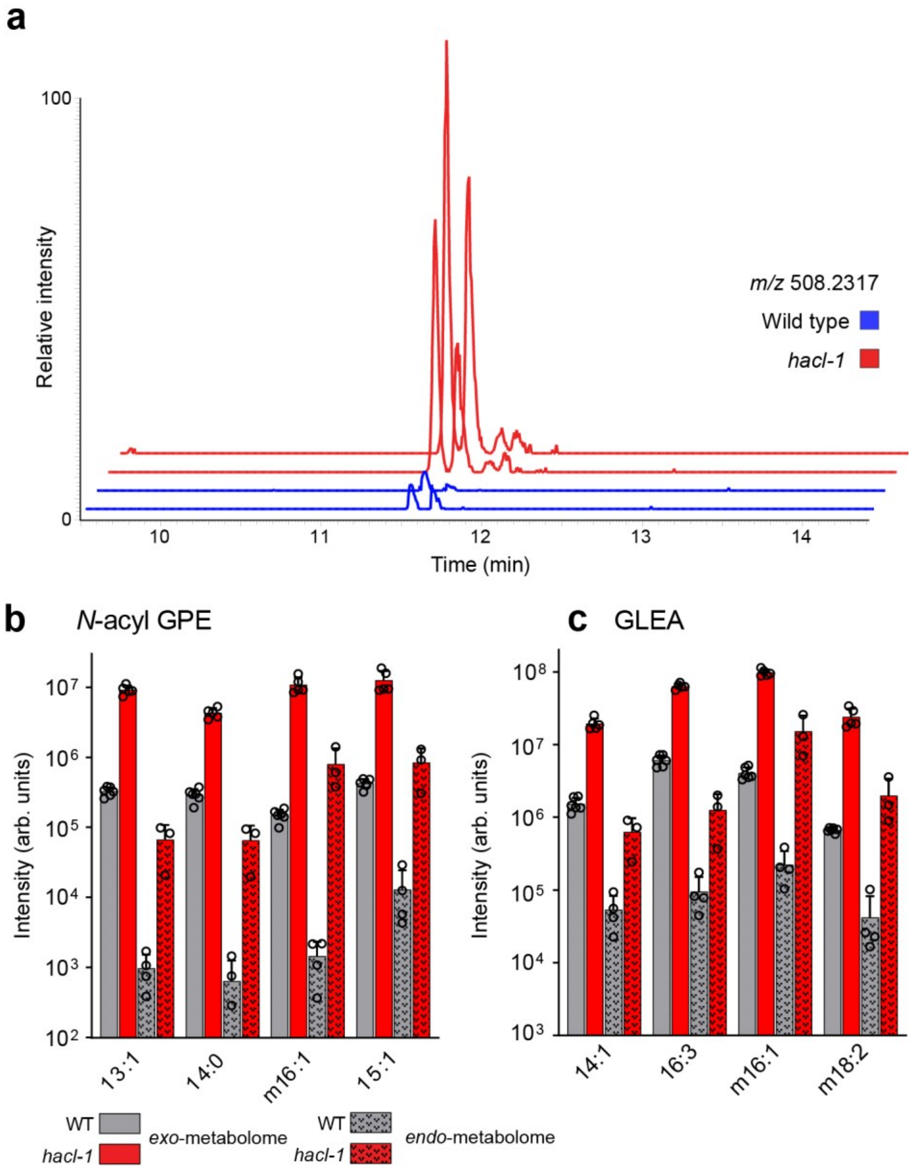

Supplementary Figure 8. *N*-acyl GPE and GLEA are more abundant in *exo*- than *endo*-metabolome. **a**, Representative HPLC-MS (negative ion) EIC for *m/z* 508.2317, corresponding to *N*-acyl GPE-14:2, in *exo*-metabolome extracts from N2 (WT) and *hacl-1*. Four distinct isomers of *N*-acyl GPE-14:2 observed (four peaks, two major, two minor). **b**, Quantification of representative *N*-acyl GPE from SN1 in the *exo*- and *endo*-metabolome extracts, as indicated. **c**, Quantification of representative GLEA from SN2 (14:1, 16:3) and SN3 (m16:1, m18:2) in the *exo*- and *endo*-metabolome extracts, as indicated. Data represent six (N2) or five (*hacl-1*) samples from three biologically independent experiments for *exo*-metabolome; data represent three biologically independent experiments for endo-metabolome and bars means ± standard deviation. Source data are provided as a Source Data file.

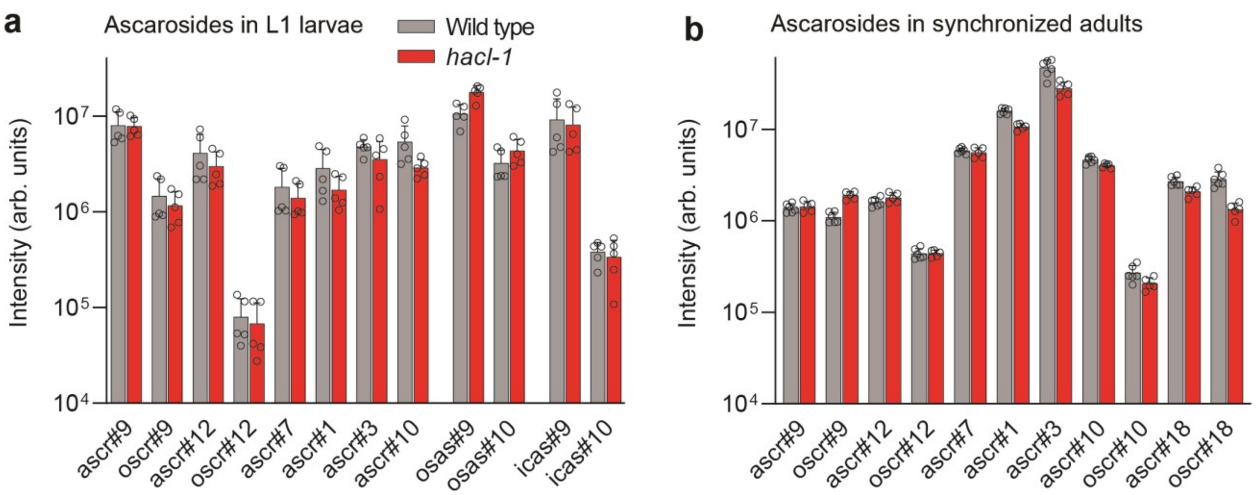

Supplementary Figure 9. Ascaroside biosynthesis is not significantly perturbed in *hacl-1* mutants. **a,** Quantification of representative ascarosides containing odd and even chain lengths from *exo*-metabolome extracts of N2 (WT) and *hacl-1* larvae, as indicated. Data represent five biologically independent experiments and bars means ± standard deviation. **b,** Quantification of representative ascarosides containing odd and even chain lengths from *exo*-metabolome extracts of N2 (WT) and *hacl-1* adults. Data represent six (N2) or five (*hacl-1*) samples from three biologically independent experiments and bars means ± standard deviation. Full structures of ascarosides listed can be accessed at the *C. elegans* Small Molecule Identifier Database (SMID-DB, www.smid-db.org). Source data are provided as a Source Data file.

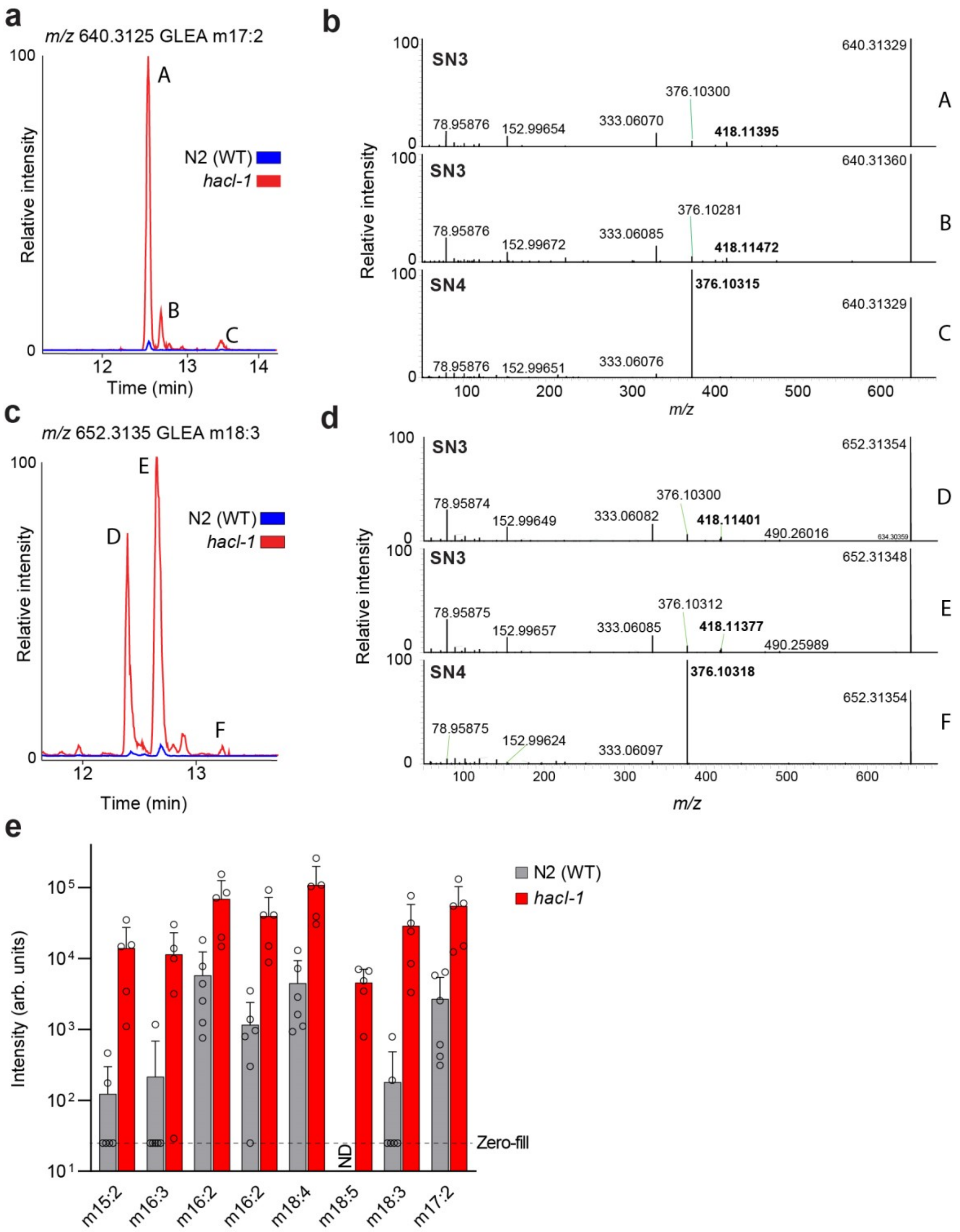

Supplementary Figure 10. GLEA in SN4 exhibit unique fragmentation and are low abundance. **a**, Representative HPLC-MS (negative ion) EIC for *m/z* 640.3125, corresponding to GLEA m17:2, which exhibits three major isomers, labeled as A, B, and C. **b,** MS/MS spectra for isomers of GLEA m17:2, as indicated in panel (a). Features A and B are networked in SN3 and produce the product ion *m/z* 418.114 during MS/MS fragmentation, whereas Feature C is networked in SN4 and produces an intense fragment ion *m/z* 376.103. **c,** Representative HPLC-MS (negative ion) EIC for *m/z* 652.3135, corresponding to GLEA m18:3, which exhibits five

major isomers, three of which are labeled as D, E, and F. **d,** MS/MS spectra for isomers of GLEA m18:3, as indicated in panel (c). The major features D and E produce product ion *m/z* 418.114 during MS/MS fragmentation and are networked in SN3, whereas F belongs to SN4 and produces an intense fragment with *m/z* 376.103. **e**, Quantification of GLEA from SN4 in *exo*-metabolome extracts of N2 (WT) and *hacl-1*, as indicated. Data represent six (N2) or five (*hacl-1*) samples from three biologically independent experiments and bars means ± standard deviation. ND, not detected. The zero-fill line from value imputation is shown in cases where a feature was not detected in a subset of samples. Source data are provided as a Source Data file.

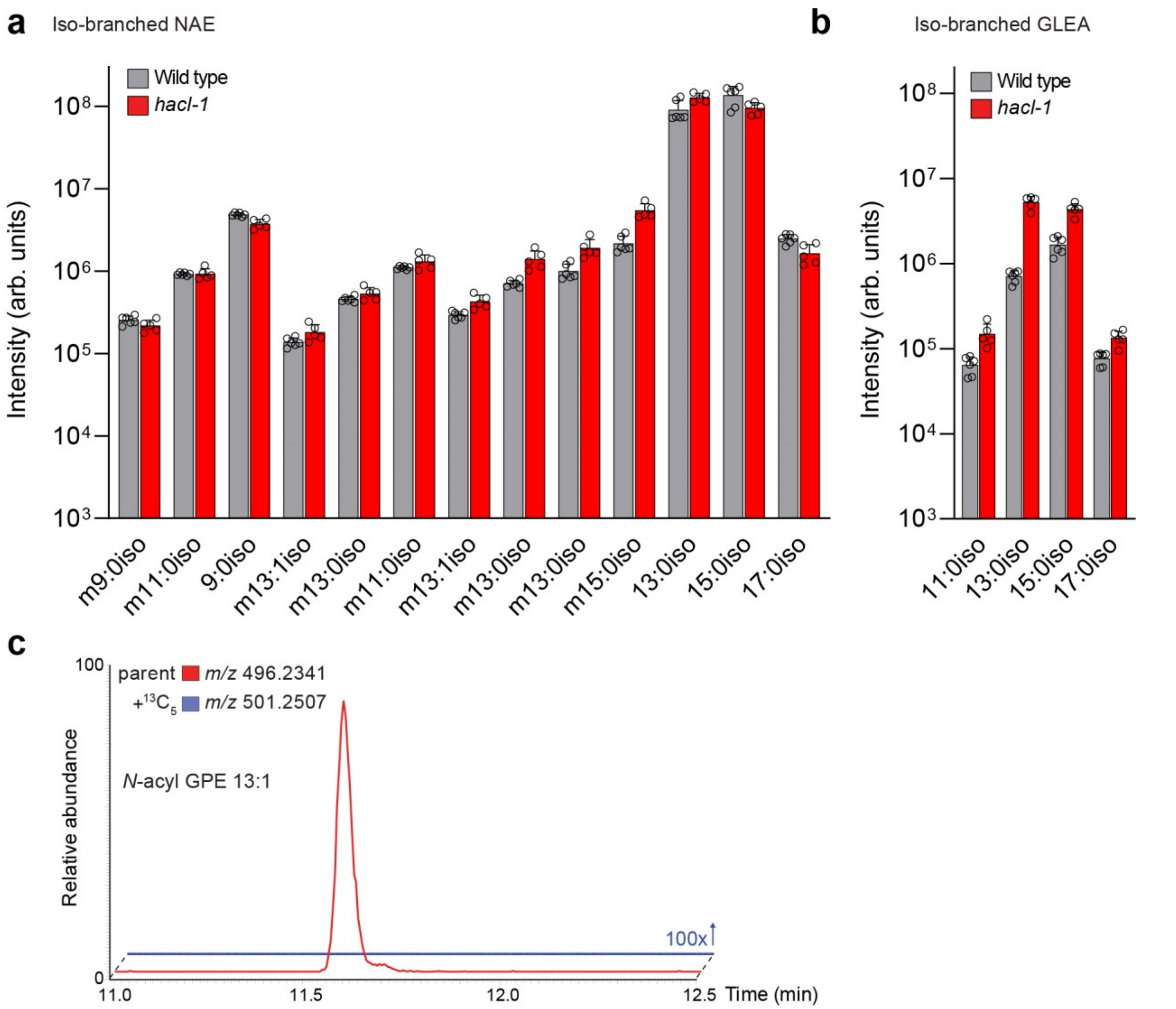

Supplementary Figure 11. Isotope tracing reveals BCFA-derived NAE and GLEA. Quantification of **a**, NAE and **b**, GLEA identified as $^{13}C_5$-enriched following $^{13}C_6$-Leu supplement from *exo*-metabolome extracts of N2 (WT) and *hacl-1* adults. Data represent six (N2) or five (*hacl-1*) samples from three biologically independent experiments and bars means ± standard deviation. **c**, Representative EICs for *m/z* 496.234 and 501.2507, corresponding to *N*-acyl GPE-13:1 and $^{13}C_5$- *N*-acyl GPE-13:1, from *exo*-metabolome extracts of N2 supplemented with $^{13}C_6$-Leu. Y-axis for *m/z* 501.2507 is scaled 100-fold to highlight absence of isotopic enrichment, demonstrating that this lipid is not a BCFA derived from Leu. Source data are provided as a Source Data file.

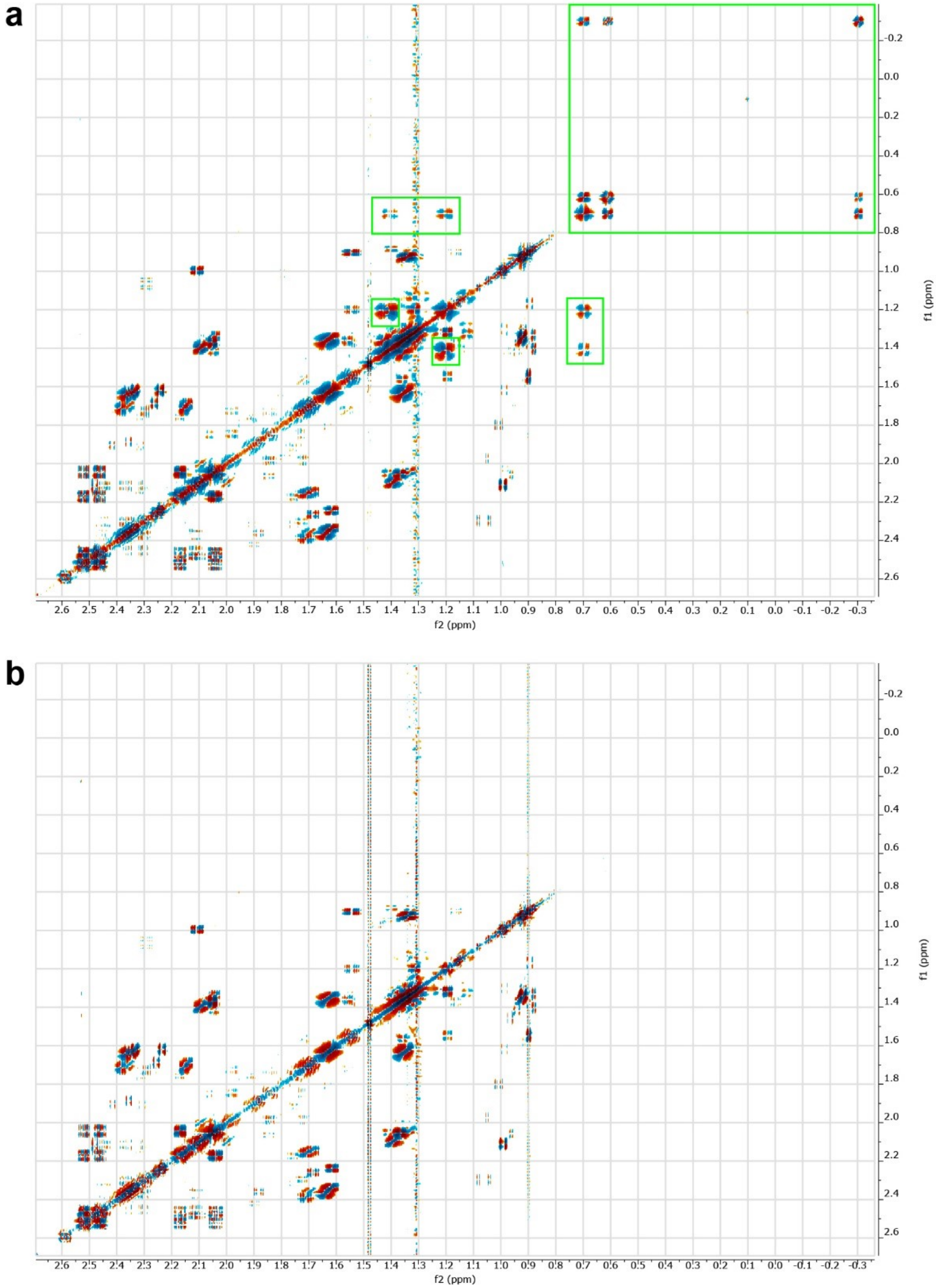

Supplementary Figure 12. JW1653-1 bacteria do not produce cyclopropane lipids. **a**, dqfCOSY spectrum (aliphatic region) of *C. elegans* reared on *E. coli* OP50 as food, showing crosspeaks characteristic for cyclopropane lipids (green boxes). **b,** dqfCOSY spectrum (aliphatic region) of *C. elegans* reared on JW1653-1 bacteria, lacking cyclopropyl signals. Spectra were acquired at 800 MHz, using CD_3OD as solvent.

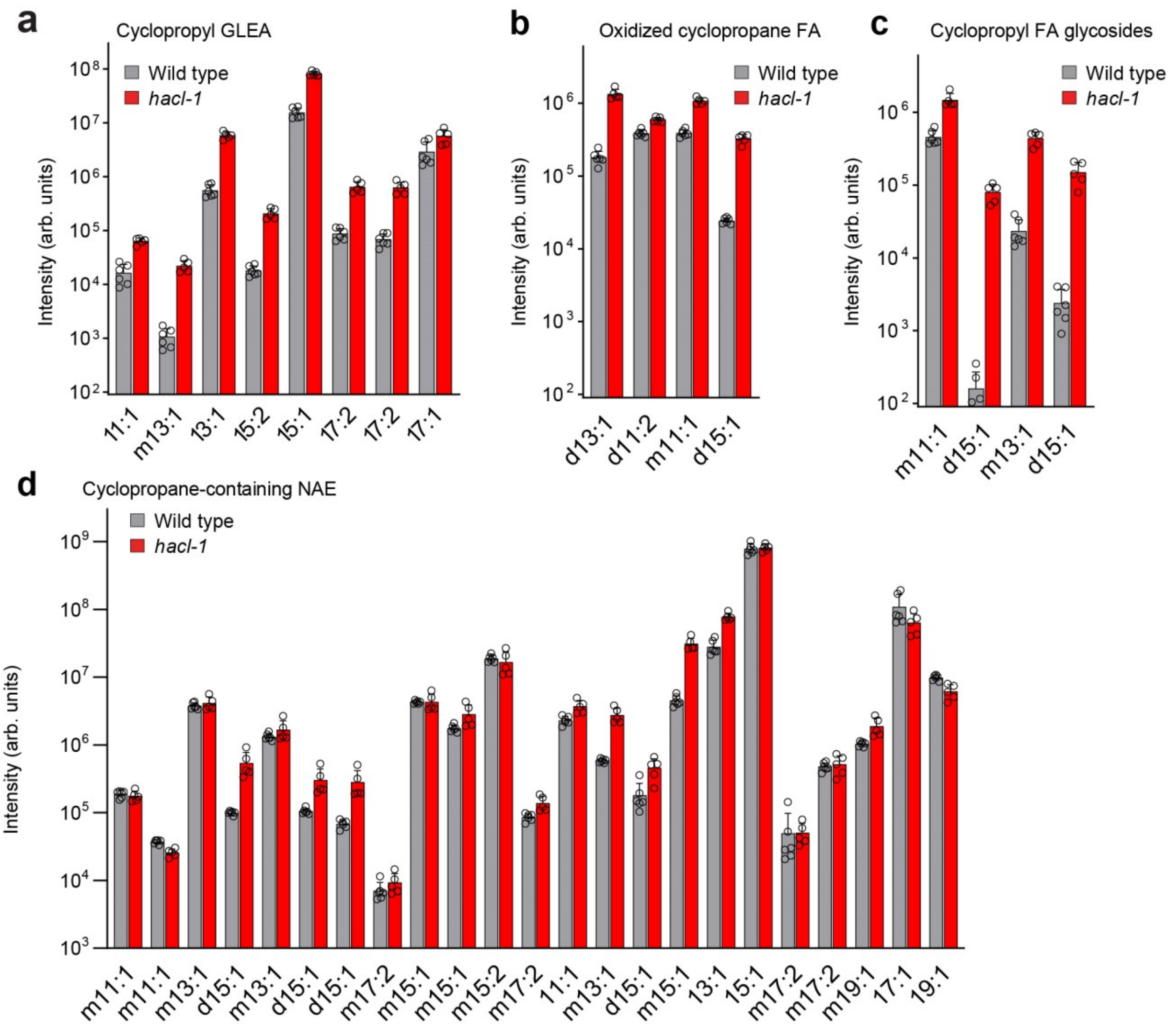

Supplementary Figure 13. Additional cyclopropane-containing metabolites identified by comparative analysis. Quantification of **a**, GLEA **b**, oxidized fatty acids **c**, fatty acyl glycosides and **d**, NAE that were absent from worms fed JW1653-1. Data represent six (N2) or five (hacl-1) samples from three biologically independent experiments and bars means ± standard deviation. Source data are provided as a Source Data file.

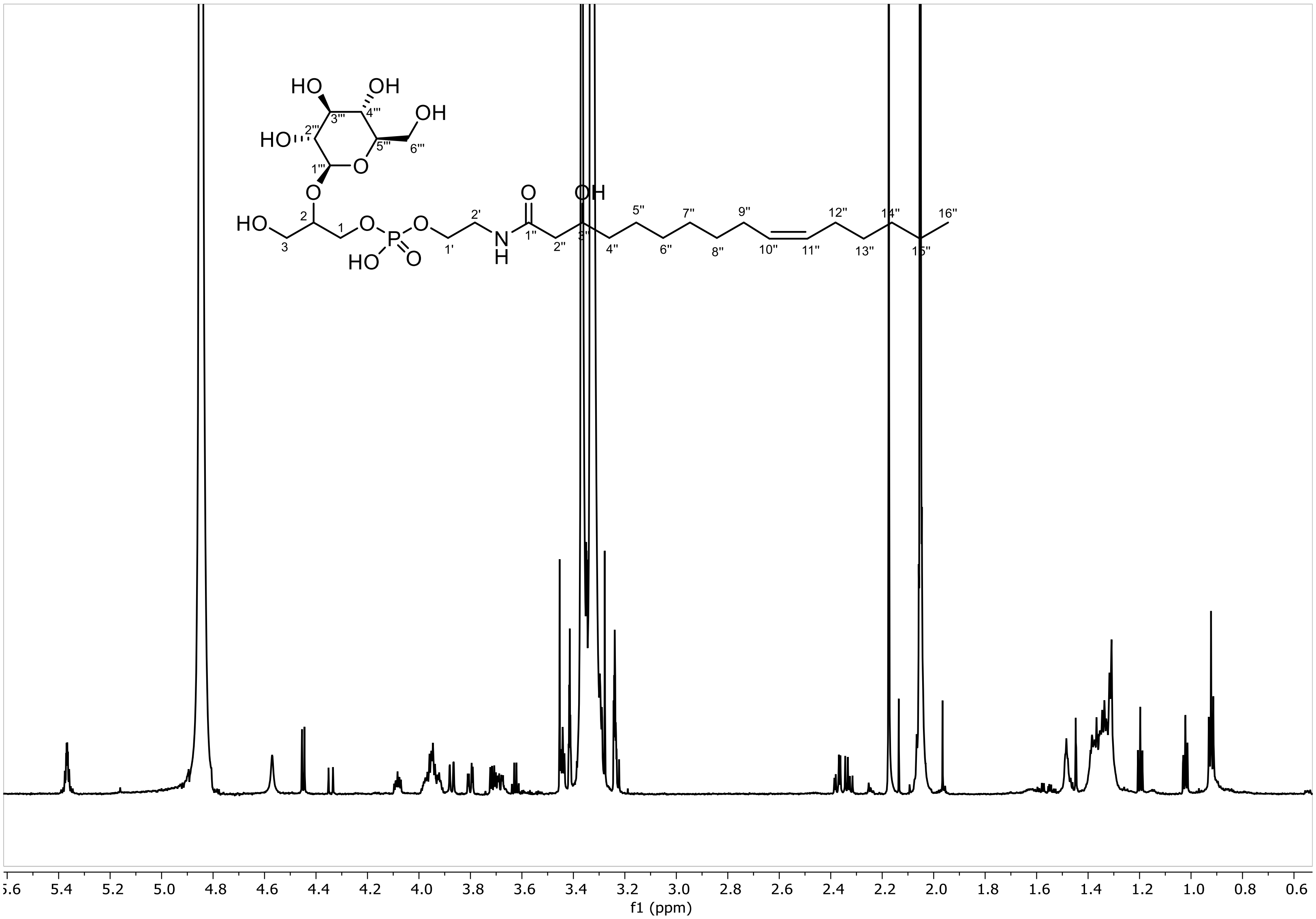

Supplementary Figure 14. ^{1}H NMR spectrum of isolated fraction containing GLEA-m16:1 (**16**), methanol-d_4 (800 MHz)

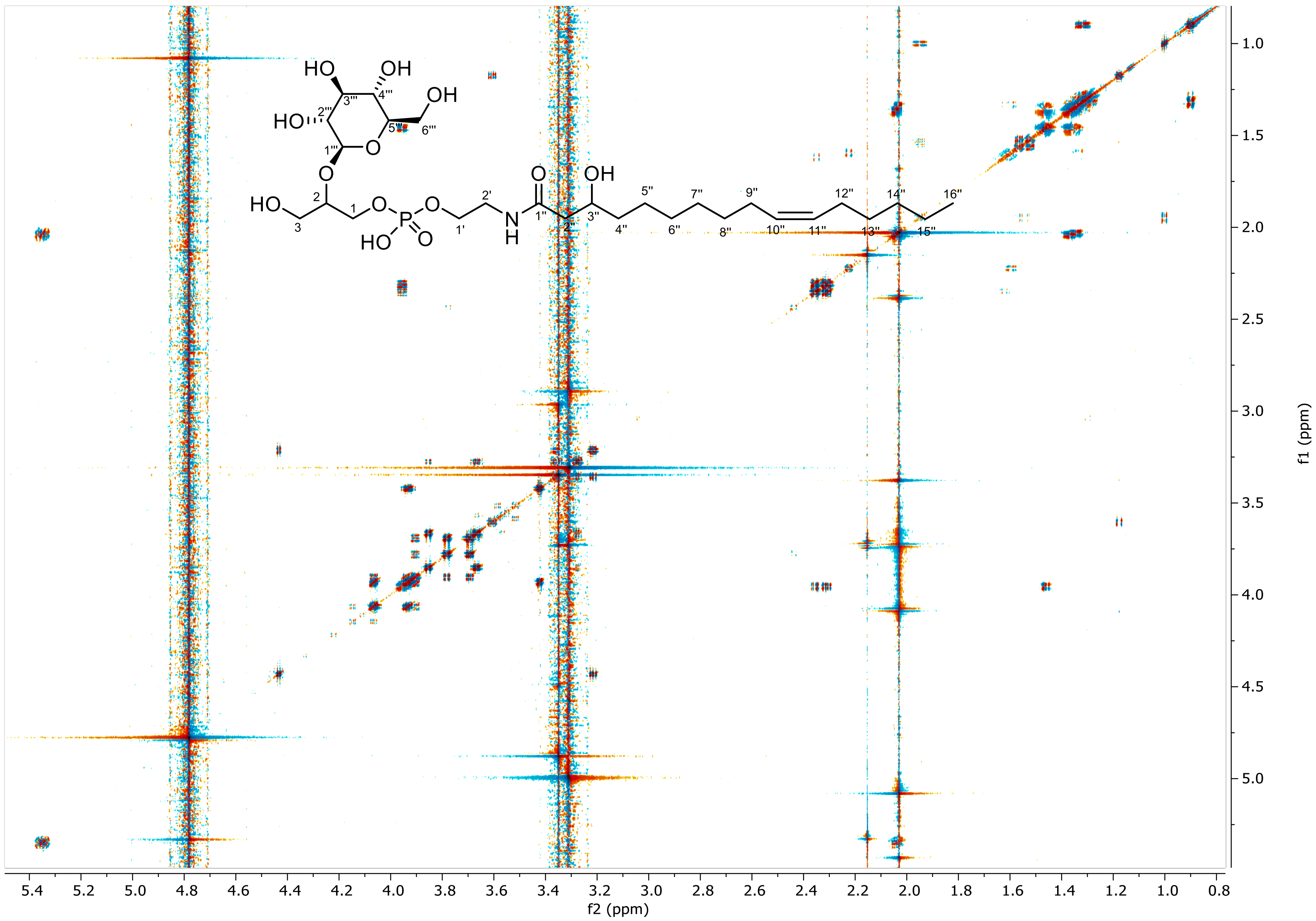

Supplementary Figure 15. dqfCOSY spectrum of metabolome fraction containing GLEA-m16:1 (**16**), methanol-d_4 (800 MHz)

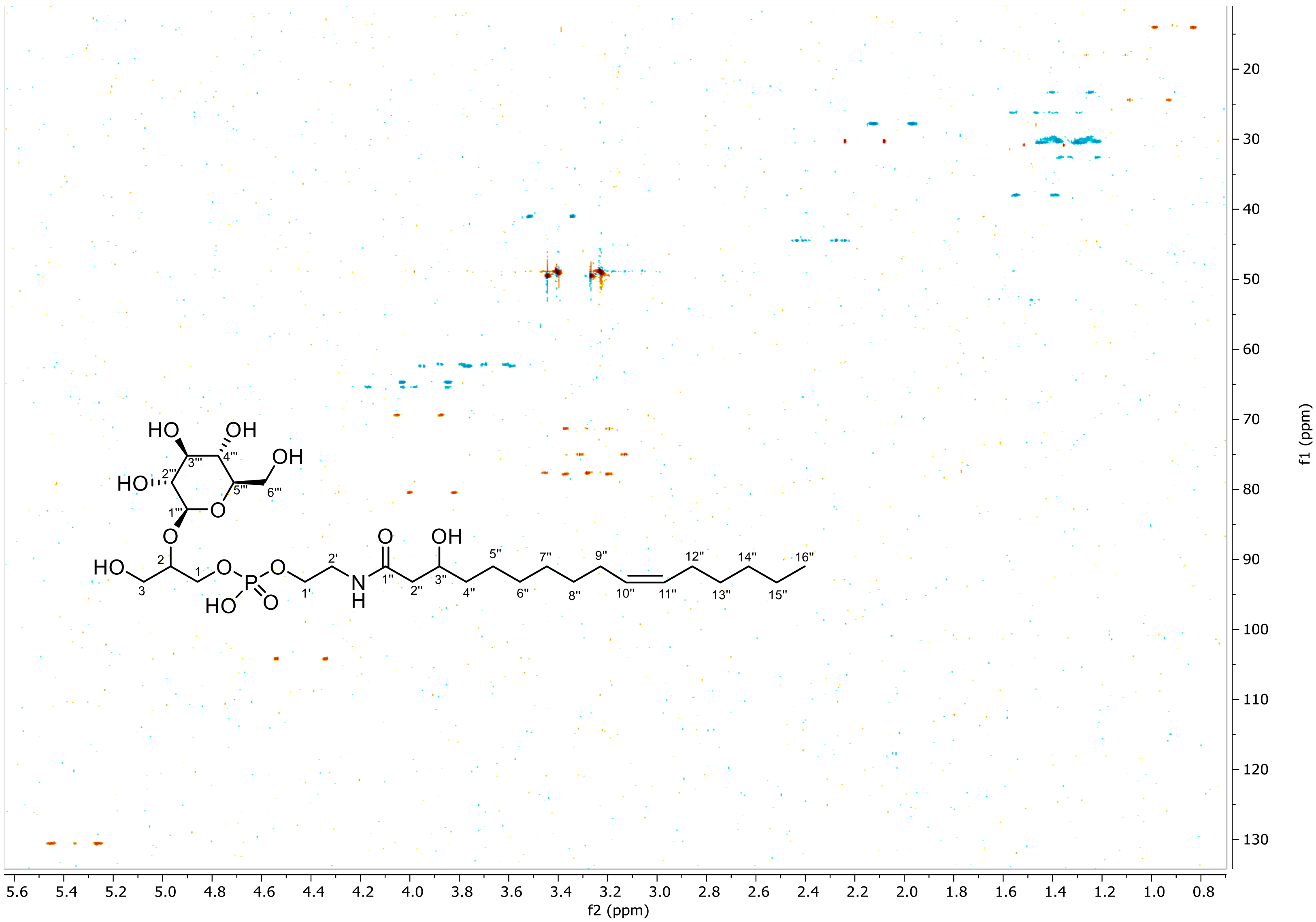

Supplementary Figure 16. HSQC spectrum of metabolome fraction containing GLEA-m16:1 (**16**), methanol-d_4 (800 MHz)

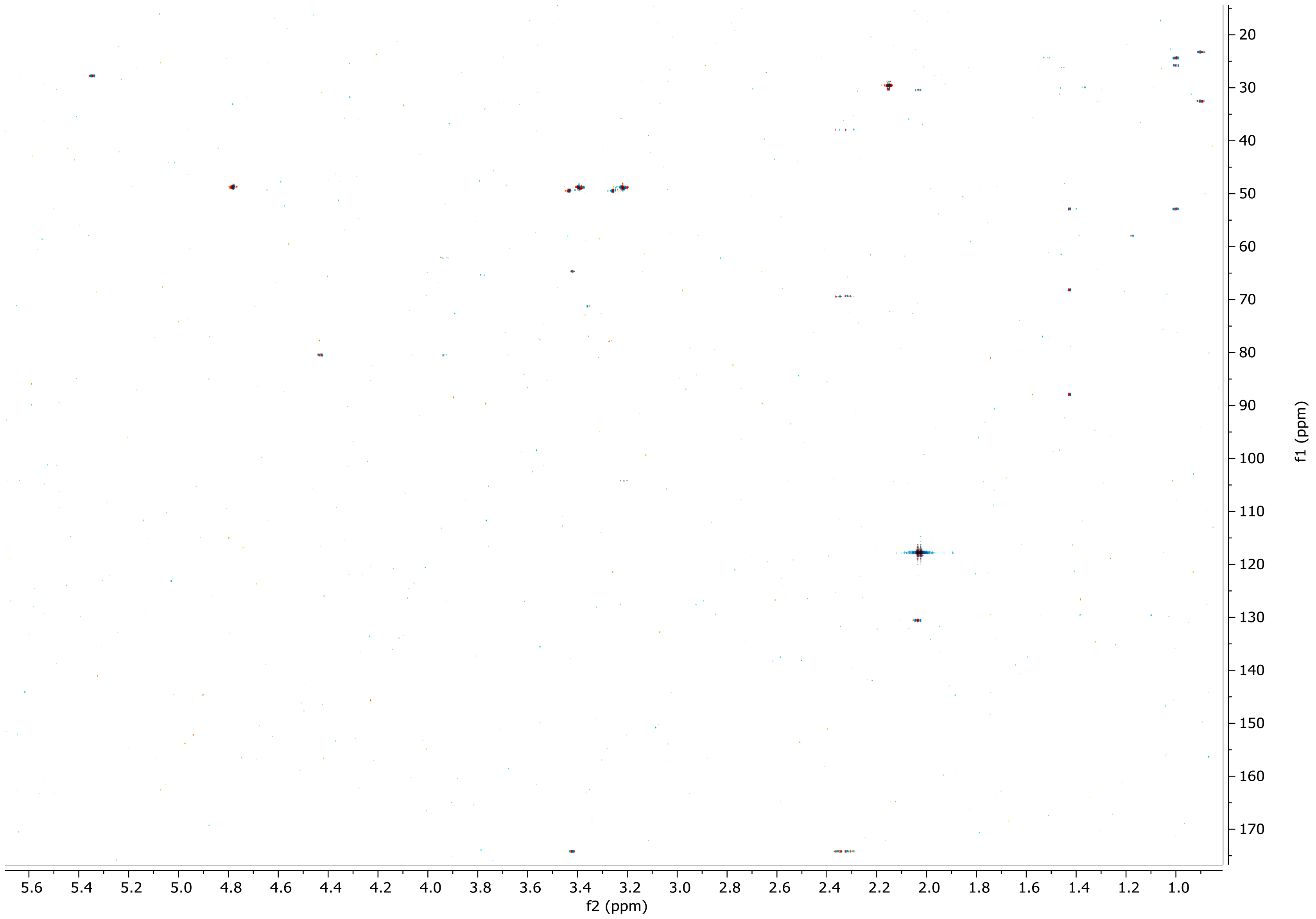

Supplementary Figure 17. HMBC spectrum of metabolome fraction containing GLEA-m16:1 (**16**), methanol-d_4 (800 MHz)

Supplementary Methods

General synthetic procedures. All oxygen and moisture-sensitive reactions were carried out under argon atmosphere in flame-dried glassware. Solutions and solvents sensitive to moisture and oxygen were transferred via standard syringe and cannula techniques. All commercial reagents were purchased as reagent grade and, unless otherwise stated, were purchased from Sigma-Aldrich and used without any further purification. Acetic acid (AcOH), acetonitrile (ACN), dichloromethane (DCM), ethyl acetate (EtOAc), formic acid, hexanes and methanol (MeOH) used for chromatography and as a reagent or solvent were purchased from Fisher Scientific. Thin-layer chromatography (TLC) was performed using J. T. Baker Silica Gel IB2F plates. Flash chromatography was performed using Teledyne Isco CombiFlash systems and Teledyne Isco RediSep Rf silica and C18 columns. All deuterated solvents were purchased from Cambridge Isotopes. Nuclear Magnetic Resonance (NMR) spectra were recorded on Bruker INOVA 500 (500 MHz) and Varian INOVA 600 (600 MHz) spectrometers at Cornell University's NMR facility and Bruker AVANCE III HD 800 MHz (800 MHz) or Bruker AVANCE III HD 600 MHz (600 MHz) at SUNY ESF's NMR facility. ^{1}H NMR chemical shifts are reported in ppm (δ) relative to residual solvent peaks (7.26 ppm for chloroform-d, 3.31 ppm for methanol-d_4). NMR-spectroscopic data are reported as follows: chemical shift, multiplicity (s = singlet, d = doublet, t = triplet, q = quartet, m = multiplet, br = broad), coupling constants (Hz). ^{13}C NMR chemical shifts are reported in ppm (δ) relative to residual solvent peaks (77.16 ppm for chloroform-d, 49.00 ppm for methanol-d_4). All NMR data processing was done using MNOVA 14.2.1 (https://mestrelab.com/).

Preparation of [Cu(*S,S*)-*tert*-butylbis(oxazolinyl)](SbF_6)$_2$

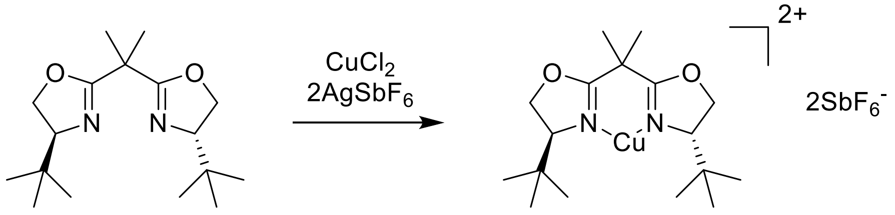

[Cu(S,S)-tert-butylbis(oxazolinyl)](SbF_6)$_2$

To a solution of (*S,S*)-2,2′-isopropylidene-bis(4-tert-butyl-2-oxazoline) (200 mg, 0.68 mmol) in dry dichloromethane (8 mL), $CuCl_2 \cdot H_2O$ (139 mg, 0.82 mmol) and $AgSbF_6$ (583 mg, 0.7 mmol) were added, and the resulting mixture was stirred at room temperature for 14 hours. The green solution was filtered through cotton for later use.

Ethyl 2-hydroxy-3-methylpent-4-enoate (14)

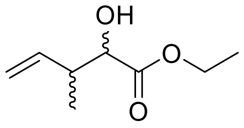

cis-butene was bubbled through a solution of ethyl glyoxylate (2 mL, 9.82 mmol) and [Cu(*S,S*)-*tert*-butylbis(oxazolinyl)](SbF_6)$_2$ at -78 °C in dry dichloromethane (2 mL), and the reaction was sealed to react at 40 °C. After 2 days, the reaction was concentrated *in vacuo*. Flash column chromatography on silica using a gradient of 0-30% ethyl acetate in hexane afforded **14** as a clear oil (977 mg, 63%, mixture of diastereomers).

Major diastereomer, ^{1}H NMR (600 MHz, chloroform-*d*): δ (ppm) 5.74 (ddd, *J* = 17.8, 9.8, 8.1 Hz, 1H), 5.07 (m, 1H), 5.05 (m, 1H), 4.24 (m, 2H), 4.10 (dd, *J* = 6.2, 3.4 Hz, 1H), 2.72 (d, *J* = 6.2 Hz, 1H), 2.69-2.61 (m, 1H), 1.29 (t, *J* = 7.2 Hz, 3H), 1.16 (d, *J* = 7.0 Hz, 3H).

Minor diastereomer, ^{1}H NMR (600 MHz, chloroform-*d*): δ (ppm) 5.85 (ddd, *J* = 17.4, 10.3, 7.4 Hz, 1H), 5.12 (m, 1H), 5.09 (m, 1H), 4.26 (m, 2H), 4.16 (dd, *J* = 6.3, 3.7 Hz, 1H), 2.76 (d, *J* = 6.3 Hz, 1H), 2.69-2.61 (m, 1H), 1.29 (t, *J* = 7.2 Hz, 3H), 1.01 (d, *J* = 7.0 Hz, 3H).

Ethyl (*E*)-2-hydroxy-3-methyldec-4-enoate (15)

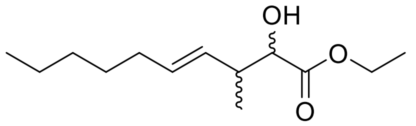

To a solution of **14** (950 mg, 6.00 mmol) dissolved in dry dichloromethane (20 mL), Grubbs catalyst 2nd generation (94 mg, 0.15 mmol) and 1-heptene (2.52 mL, 18.00 mmol) were added at room temperature. The reaction mixture was allowed to stir 20 h and was concentrated *in vacuo*. Flash column chromatography on silica using a gradient of 0-30 % ethyl acetate in hexanes afforded **15** as a clear oil (785 mg, 76% BRSM, mixture of diastereomers).

Major diastereomer, ^{1}H NMR (600 MHz, chloroform-*d*): δ (ppm) 5.46 (dt, *J* = 15.4, 6.7 Hz, 1H), 5.31 (dd, *J* = 15.4, 8.2 Hz, 1H), 4.27-4.18 (m, 2H), 4.06 (m, 1H), 2.67 (d, *J* = 5.2 Hz, 1H), 2.60 (m, 1H), 1.96 (q, *J* = 7.3 Hz, 2H), 1.36-1.20 (m, 6H), 1.29 (t, *J* = 7.3 Hz, 3H), 1.13 (d, *J* = 7.1 Hz, 3H), 0.87 (t, *J* = 7.2 Hz, 3H).

Minor diastereomer, ^{1}H NMR (600 MHz, chloroform-*d*): δ (ppm) 5.52 (dt, *J* = 15.4, 6.5 Hz, 1H), 5.40 (dd, *J* = 15.4, 7.8 Hz, 1H), 4.27-4.18 (m, 2H), 4.09 (m, 1H), 2.70 (d, *J* = 6.8 Hz, 1H), 2.56 (m, 1H), 2.00 (q, *J* = 7.2 Hz, 2H), 1.36-1.20 (m, 6H), 1.28 (t, *J* = 7.2 Hz, 3H), 1.12 (d, *J* = 7.0 Hz, 3H), 0.87 (t, *J* = 7.1 Hz, 3H).

(*E*)-2-hydroxy-3-methyldec-4-enoic acid (bemeth#2, 12)

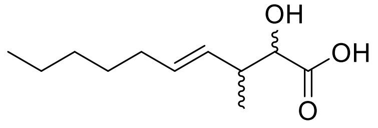

To a solution of **15** (76 mg, 0.33 mmol) in 1,4-dioxane (2 mL), lithium hydroxide monohydrate (42 mg, 1.00 mmol) in H_2O (1 mL) was added, and the resulting mixture was stirred at 60 °C for 12 hours. Glacial acetic acid (0.5 mL) was added, and the reaction was concentrated *in vacuo*. Flash column chromatography on silica using a gradient of 0-50% ethyl acetate in hexanes afforded **12** (46.7 mg, 70%) as a clear oil.

For ^{1}H and ^{13}C NMR spectroscopic data of the major diastereomer of bemeth#2 (**12)**, see **Supplementary Table 3**.

Minor diastereomer, ^{1}H NMR (600 MHz, methanol-*d*$_4$): δ (ppm) δ (ppm) 5.56-5.41 (m, 2H), 3.96 (d, *J* = 4.6 Hz, 1H), 2.54 (m, 1H), 2.00 (m, 2H), 1.43-1.25 (m, 6H), 1.00 (d, *J* = 6.9 Hz, 3H), 0.90 (t, *J* = 7.0 Hz, 3H).

Supplementary Tables

Supplementary Table 1. Comparison of Metaboseek to other free, open-source metabolomics tools with graphical user interfaces (GUI). Metaboseek offers flexibility in deployment as well as a unique combination of functionalities for discovery metabolomics.

	Metaboseek	MZmine2	XCMS online	MS-DIAL	GNPS Dashboard	MetaboAnalyst
**Deployment**						
Server / local	Both	Local	Online	Local	Online	Online
Open-Source	+	+	Partial	+	+	Partial
Language	R/shiny	Java	R/Java script	C#	Python	R/PrimeFaces
**Raw Data Processing**						
Feature detection	+	+	+	+	Limited	+
Interactive raw data browser	+	+	-	+	+	-
Grouped EIC plot	+	-	Limited	+	-	-
**Feature Tables**						
Import feature tables	+	+	-	-	-	+
Statistical analysis	+	+	+	+	-	+
Custom sample group filters	+	-	-	-	-	-
**Structural Characterization**						
Formula prediction / database lookup	+	+	+	+	+	+
Molecular Networking	+	Export	Export	+	+	-
SIRIUS fragment annotation	+	+	-	-	-	-
MS2 pattern search	+	+	-	+	-	-
Isotope Tracking	+	-	-	+	-	-

Supplementary Table 2. NMR spectroscopic data of bemeth#3 (11) (600 MHz, methanol-d_4).

Position	δ ^{1}H [ppm]	^{1}H-^{1}H-coupling constants [Hz]
1		
2	3.84	$J_{2,3}$ = 3.8 Hz
3	2.60	$J_{2,3}$ = 8.5 Hz
4	5.53	$J_{4,5}$ = 16 Hz
5	5.50	$J_{5,6}$ = 7.3 Hz
6	2.02	
7	1.40	
	1.48	
8	1.41	
	1.47	
9	3.72	$J_{9,10}$ = 6.3
10	1.17	
3-CH_3	0.92	$J_{3,3\text{-}CH3}$ = 6.9

Supplementary Table 3. NMR spectroscopic data of bemeth#2 (12), major diastereomer (600 MHz, methanol-d_4).

Position	δ ^{13}C [ppm]	δ ^{1}H [ppm]	^{1}H-^{1}H-coupling constants [Hz]	HMBC correlations
1	179.3			
2	76.8	3.86	$J_{2,3}$ = 3.8 Hz	C-1 (weak), C-11
3	41.5	2.59		
4	134.4	5.50		C-3, C-6, C-11
5	130.7	5.49		C-3, C-6
6	33.4	2.00	$J_{5,6}$ = 7.3, $J_{6,7}$ = 6.0	C-4, C-5, C-7, C-8
7	30.1	1.38		C-5, C-6, C-8, C-9
8	32.3	1.31		C-6, C-7, C-9, C-10
9	23.4	1.32	$J_{9,10}$ = 7.1	C-7, C-8, C-10
10	14.1	0.90		C-8, C-9
3-CH_3	14.2	0.92	$J_{3,3\text{-}CH3}$ = 6.9	C-2, C-3, C-4

Supplementary Table 4. NMR spectroscopic data for GLEA-m16:1 (**16**), methanol-d_4 (800 MHz).

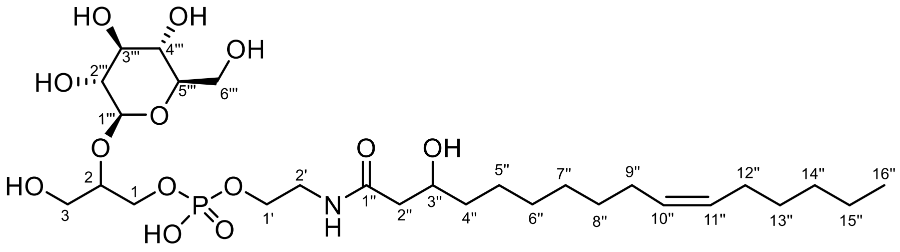

16 Proposed structure, position of double bond not known

Position	δ ^{13}C [ppm]	δ ^{1}H [ppm] (J_{HH}/J_{HP} [Hz])	Key HMBC signals
1”	174.2		
2”a	44.5	2.31 (dd, $J_{2a'',2b''}$ = 15.0, $J_{2a'',3''}$ = 8.1)	C-1”
2”b		2.36 (dd, $J_{2b'',3''}$ = 4.4)	C-1”
3”	69.4	3.96 (m)	
4”	38.0	1.46 (m, 2 H)	
5”-8”	29.8-30.7 (4 C)	1.32-1.38 (m, 8 H)	
9”/12”	27.8	2.02-2.06 (m, 4 H)	
10”/11”	130.5 (2C)	5.33-5.36 (m, 2H)	
13”-15”	29.8-30.7 (3 C)	1.29-1.38 (m, 6 H)	
16”	14.1	0.90 (t, $J_{15'',16''}$ = 7.0)	
1′	64.7	3.93 (dt, $J_{1',P}$ = 6.5, $J_{1',2'}$ = 5.8)	
2′	41.1	3.42 (t, $J_{1',2'}$ = 5.8)	C-1”
1a	65.4	3.93 (ddd, $J_{1a,2}$ = 6.8, $J_{1a,1b}$ = 10.7, $J_{1a,P}$ = 6.3)	
1b		4.06 (ddd, $J_{1b,2}$ = 3.8, $J_{1b,P}$ = 6.3)	
2	80.5	3.90 (m)	C-1’’’ (weak)
3a	62.2	3.69 (dd, $J_{2,3a}$ = 6.0, $J_{3a,3b}$ = 12.5)	
3b		3.78 (dd, $J_{2,3b}$ = 3.4)	
1‴	104.2	4.43 (d, $J_{1''',2'''}$ = 8)	C-2
2‴	75.0	3.22 (dd, $J_{3''',4'''}$ = 9)	C-1’’’
3‴	77.6	3.36 (m)	
4‴	71.3 or 77.9	3.26-3.29 (m)	
5‴	71.3 or 77.9	3.26-3.29 (m)	
6a‴	62.4	3.67 (dd, $J_{5''',6a'''}$ = 5.6, $J_{6a''',6b'''}$ = 12)	
6b‴		3.85 (dd, $J_{5''',6b'''}$ = 2)	
