# Supplementary material for: Comparative metabolomics with Metaboseek reveals functions of a conserved fat metabolism pathway in C. elegans
Source: Nat Commun. 2022 Feb 10;13:782. doi: 10.1038/s41467-022-28391-9 (PMC8831614; doi:10.1038/s41467-022-28391-9)
Supplement: Supplementary file 3 — Description of Additional Supplementary Files [file 41467_2022_28391_MOESM3_ESM.docx]

**Comparative metabolomics with Metaboseek reveals functions
of a conserved fat metabolism pathway in *C. elegans***

**Description of Additional Supplementary Files**

Maximilian J. Helf^1^, Bennett W. Fox^1^, Alexander B. Artyukhin^2^, Ying K. Zhang^1^, Frank C. Schroeder^1,^*

^1^Boyce Thompson Institute and Department of Chemistry and Chemical Biology, Cornell University, Ithaca, New York 14853, United States, ^2^Chemistry Department, College of Environmental Science and Forestry, State University of New York, Syracuse, New York 13210, United States

These authors contributed equally: Maximilian J. Helf, Bennett W. Fox

*Correspondence to [fs31@cornell.edu](mailto:fs31@cornell.edu)

File Name: Supplementary Data 1

Description: Tabulated feature numbers remaining after background subtraction at different *Fast Peak Shapes* (FPQ) peak quality thresholds in negative and positive ionization modes of *exo*-metabolome extract, as indicated. Features less than ten-fold more abundant in *C. elegans* samples relative to blanks were omitted. Following background subtraction, data was grouped according to genotype and analyzed for 5-fold enrichment of *hacl-1* over N2 (WT) at different mean intensity thresholds.

File Name: Supplementary Data 2

Description: Annotated table of both known and novel metabolites identified in the *exo*-metabolome. Values from Metaboseek include *m/z*, *m/z* range, retention time (rt), rt range, mean intensity of samples grouped according to genotype, fold-change of *hacl-1* relative to N2 (WT), and unadjusted *p-*value calculated by two-tailed unpaired *t-*test in Metaboseek. Additional manually annotated columns include predicted uncharged molecular formulae, tentative compound class assignment, composition of acyl group (where applicable), Small Molecule Identifier Database (SMID-DB) unique metabolite name (where applicable), and a comment i) to provide additional description relevant to the main findings (e.g., if a metabolite likely incorporates a cyclopropyl moiety or was isotopically enriched following ^13^C_6_-Leu supplement), ii) to indicate that the major species was detected as an adduct, or iii) to indicate issues with rt drift or peak picking (where indicated).
