# Supplementary material for: Comparative metabolomics with Metaboseek reveals functions of a conserved fat metabolism pathway in C. elegans
Source: Nat Commun. 2022 Feb 10;13:782. doi: 10.1038/s41467-022-28391-9 (PMC8831614; doi:10.1038/s41467-022-28391-9)
Supplement: Supplementary file 6 — Reporting Summary [file 41467_2022_28391_MOESM6_ESM.pdf]

nature portfolio

Corresponding author(s): Frank Schroeder

Last updated by author(s): Jan 7, 2022

Reporting Summary

Nature Portfolio wishes to improve the reproducibility of the work that we publish. This form provides structure for consistency and transparency in reporting. For further information on Nature Portfolio policies, see our Editorial Policies and the Editorial Policy Checklist.

Statistics

For all statistical analyses, confirm that the following items are present in the figure legend, table legend, main text, or Methods section.

n/a	Confirmed	
☐	☒	The exact sample size (*n*) for each experimental group/condition, given as a discrete number and unit of measurement
☐	☒	A statement on whether measurements were taken from distinct samples or whether the same sample was measured repeatedly
☐	☒	The statistical test(s) used AND whether they are one- or two-sided<br>*Only common tests should be described solely by name; describe more complex techniques in the Methods section.*
☐	☒	A description of all covariates tested
☐	☒	A description of any assumptions or corrections, such as tests of normality and adjustment for multiple comparisons
☐	☒	A full description of the statistical parameters including central tendency (e.g. means) or other basic estimates (e.g. regression coefficient) AND variation (e.g. standard deviation) or associated estimates of uncertainty (e.g. confidence intervals)
☐	☒	For null hypothesis testing, the test statistic (e.g. *F*, *t*, *r*) with confidence intervals, effect sizes, degrees of freedom and *P* value noted<br>*Give P values as exact values whenever suitable.*
☒	☐	For Bayesian analysis, information on the choice of priors and Markov chain Monte Carlo settings
☒	☐	For hierarchical and complex designs, identification of the appropriate level for tests and full reporting of outcomes
☒	☐	Estimates of effect sizes (e.g. Cohen's *d*, Pearson's *r*), indicating how they were calculated

Our web collection on statistics for biologists contains articles on many of the points above.

Software and code

Policy information about availability of computer code

Data collection	MS data acquisition was controlled by Chromeleon software (Thermo Scientific v7.3) and Xcalibur software (Thermo Scientific v4.1.31.9).
Data analysis	RAW data files were converted to mzXML file format using MSConvert (v3.0, ProteoWizard). Data was analyzed using Metaboseek software (current v0.9.9) available here: [https://doi.org/10.5281/zenodo.3360087] and Xcalibur Quan Browser (Thermo Scientific v4.1.31.9).

For manuscripts utilizing custom algorithms or software that are central to the research but not yet described in published literature, software must be made available to editors and reviewers. We strongly encourage code deposition in a community repository (e.g. GitHub). See the Nature Portfolio guidelines for submitting code & software for further information.

Data

Policy information about availability of data

All manuscripts must include a data availability statement. This statement should provide the following information, where applicable:

- Accession codes, unique identifiers, or web links for publicly available datasets
- A description of any restrictions on data availability
- For clinical datasets or third party data, please ensure that the statement adheres to our policy

The HPLC-HRMS data generated during this study have been deposited in the MassIVE database under accession code MSV000087885 [https://doi:10.25345/C5P24X]. Source data are provided with this paper. Detailed information about ascarosides and other known C. elegans compounds can be accessed via the Small Molecule Identifier Database (SMID-DB, https://www.smid-db.org/).

Field-specific reporting

Please select the one below that is the best fit for your research. If you are not sure, read the appropriate sections before making your selection.

☒ Life sciences ☐ Behavioural & social sciences ☐ Ecological, evolutionary & environmental sciences

For a reference copy of the document with all sections, see nature.com/documents/nr-reporting-summary-flat.pdf

Life sciences study design

All studies must disclose on these points even when the disclosure is negative.

Sample size	No sample size calculations were performed. The sample sizes in the study were chosen based on prior experience and reasonable replication.<br><br>- For larvae, more than ten independent biological experiments were conducted by two different people, for which a subset were chosen as representative. In the representative experiments, animals were treated with vehicle or supplemented with branched fatty acids, resulting in analysis of five samples for each genotype.<br>- For synchronized adult animals, three independent biological experiments were conducted, in which two technical replicates for each genotype were grown in parallel, with technical replicates extracted in two independent groups. One experiment contained only one replicate for the mutant (hacl-1), so the total number of samples was six and five, respectively.<br>- For isotope labeling experiments, two independent experiments were performed with the WT genotype in which animals were supplemented with light or heavy leucine, respectively.<br>- For modified diet experiments (cyclopropane-deficient E. coli), three (hacl-1) and two (WT) independent experiments were performed in which animals were fed the reference diet or modified diet in parallel.
Data exclusions	No data were excluded.
Replication	Experiments were conducted independently by two different people (BWF and ABA) over the course of multiple years. Attempts at reproduction were successful.
Randomization	Samples were allocated into experimental groups based on genotype, supplement, and/or diet. In the majority of experiments, WT and hacl-1 mutant were grown in parallel. In any experiment investigating supplement or modified diet, animals were grown in parallel under reference conditions.
Blinding	Blinding was not possible during data collection because the acquired experimental data required manipulations of live animals. However, data analysis was fully automated.

Reporting for specific materials, systems and methods

We require information from authors about some types of materials, experimental systems and methods used in many studies. Here, indicate whether each material, system or method listed is relevant to your study. If you are not sure if a list item applies to your research, read the appropriate section before selecting a response.

Materials & experimental systems

n/a	Involved in the study	
☒	☐	Antibodies
☒	☐	Eukaryotic cell lines
☒	☐	Palaeontology and archaeology
☐	☒	Animals and other organisms
☒	☐	Human research participants
☒	☐	Clinical data
☒	☐	Dual use research of concern

Methods

n/a	Involved in the study	
☒	☐	ChIP-seq
☒	☐	Flow cytometry
☒	☐	MRI-based neuroimaging

Animals and other organisms

Policy information about studies involving animals; ARRIVE guidelines recommended for reporting animal research

Laboratory animals	The C. elegans laboratory strain (N2 Bristol) and deletion mutant B0334.3/hacl-1, allele tm6725, strain designation FCS7. C. elegans are predominantly self-fertilizing hermaphrodites grown for between 1-3 days prior to harvest.
Wild animals	No wild animals were used in this study.
Field-collected samples	No field-collected samples were used in this study.
Ethics oversight	No ethical approval was required.

Note that full information on the approval of the study protocol must also be provided in the manuscript.
